# Evolution of *Mycobacterium tuberculosis* complex lineages and their role in an emerging threat of multidrug resistant tuberculosis in Bamako, Mali

**DOI:** 10.1038/s41598-019-56001-0

**Published:** 2020-01-15

**Authors:** Madikay Senghore, Bassirou Diarra, Florian Gehre, Jacob Otu, Archibald Worwui, Abdul Khalie Muhammad, Brenda Kwambana-Adams, Gemma L. Kay, Moumine Sanogo, Bocar Baya, Susan Orsega, Seydou Doumbia, Souleymane Diallo, Bouke C. de Jong, Mark J. Pallen, Martin Antonio

**Affiliations:** 1 Medical Research Council Unit The Gambia at The London School of Hygiene & Tropical Medicine, Atlantic Boulevard, Fajara, PO Box 273 Banjul The Gambia; 2 Division of Microbiology & Immunity, Warwick Medical School, University of Warwick, Coventry, CV4 7AL UK; 3 University Clinical Research Center (UCRC)-SEREFO-Laboratory, University of Sciences, Techniques and Technologies of Bamako (USTTB), Bamako, Mali; 4 Institute of Tropical Medicine, Antwerp, Belgium; 5 Norwich Medical School, University of East Anglia, Norwich, NR4 7TJ UK; 6 Collaborative Clinical Research Branch, Division of Clinical Research, National Institute of Allergy and Infectious Diseases, Bethesda, Maryland USA; 7 Quadram Institute, Norwich Research Park, Norwich, Norfolk NR4 7UA UK

**Keywords:** Phylogenetics, Bacterial genomics

## Abstract

In recent years Bamako has been faced with an emerging threat from multidrug resistant TB (MDR-TB). Whole genome sequence analysis was performed on a subset of 76 isolates from a total of 208 isolates recovered from tuberculosis patients in Bamako, Mali between 2006 and 2012. Among the 76 patients, 61(80.3%) new cases and 15(19.7%) retreatment cases, 12 (16%) were infected by MDR-TB. The dominant lineage was the Euro-American lineage, Lineage 4. Within Lineage 4, the Cameroon genotype was the most prevalent genotype (n = 20, 26%), followed by the Ghana genotype (n = 16, 21%). A sub-clade of the Cameroon genotype, which emerged ~22 years ago was likely to be involved in community transmission. A sub-clade of the Ghana genotype that arose approximately 30 years ago was an important cause of MDR-TB in Bamako. The Ghana genotype isolates appeared more likely to be MDR than other genotypes after controlling for treatment history. We identified a clade of four related Beijing isolates that included one MDR-TB isolate. It is a major concern to find the Cameroon and Ghana genotypes involved in community transmission and MDR-TB respectively. The presence of the Beijing genotype in Bamako remains worrying, given its high transmissibility and virulence.

## Introduction

Tuberculosis is caused by members of the *Mycobacterium tuberculosis* complex (MTBC). In humans, the main causes include *Mycobacterium tuberculosis sensu stricto* and *Mycobacterium africanum*^1^. *M. tuberculosis* and *M. africanum* form seven distinct phylogenetic lineages that are believed to have co-evolved with humans over millennia^2–4^. The East Asian, East-African-Indian and Euro-American lineages (Lineages 2,3 and 4 respectively) form the “modern” clade of tuberculosis^5^. The East Asian (including the Beijing genotype) and Euro-American lineages are the most widespread lineages globally and are probably more virulent than other lineages of the MTBC^6^. Recently a new lineage (lineage 7) has been discovered, which is predominantly restricted to Ethiopia in the horn of Africa^7,8^.

A unique feature of TB in West Africa is the presence of six major lineages (Lineages 1–6). The Euro-American (Lineage 4) and the two *M. africanum* lineages (Lineages 5 and 6) are the most common causes of human pulmonary TB in West Africa^9^. The Cameroon genotype of Lineage 4 (also known as LAM-10) is dominant in much of West Africa^10–14^, although the Ghana genotype of Lineage 4, which often have spoligo patterns that belong to T1 clade, is also disseminated across almost all West African countries^9^. The two *M. africanum* lineages are generally restricted to West Africa and cases outside the region are often linked to West African migrants^9^. Although the prevalence of Beijing strains (lineage 2) is low in West Africa a high prevalence has been reported in Benin and Senegal, prompting concerns about its emergence in the region^15,16^.

Despite the availability of effective treatment, tuberculosis (TB) remains one of the leading causes of death from infectious disease globally. In 2015, the WHO estimated that there were over ten million new cases of TB globally and 1.5 million deaths^17^. In sub-Saharan Africa, the fight against TB faces unique challenges due to the combination of the HIV epidemic, the emergence of multidrug-resistant (MDR-TB) strains and inadequate infrastructure for TB case management^18,19^. A drug resistance survey of MTBC drug resistance in West Africa was conducted between 2008 and 2013 by the West African Nodes of Excellence for AIDS Tuberculosis and Malaria (WANETAM). The survey showed that the estimated prevalence of MDR-TB in West Africa was likely being underestimated and called for urgent measure to tackle the growing threat from MDR-TB in West Africa, one of the poorest regions in the world^20^.

Bamako, the capital city of Mali, is one of few West African cities that have a well-structured hierarchy for TB case management. However, in recent years, Bamako has been faced with an emerging threat from MDR-TB^20,21^. Between 2006 and 2014 a surveillance was carried out in Bamako, which included 522 patients treated for pulmonary tuberculosis Bamako^22^. Phenotypic drug susceptibility testing on 337 unique isolates of MTBC showed resistance to at least one drug was found in 127 (37.7%) of which 75 (22.3%) were multidrug resistance (MDR)^22^. Spoligotyping confirmed that the most prevalent genotypes over the ten-year study period were MTB T1 (31.9%) and MTB LAM10 (Cameroon Genotype) (15.3%) from lineage 4 and *M. africanum* 2 (16.8%)^23^. These proportions were similar to results published by Traore *et al*. in 2012 where he reported that the most prevalent genotypes causing tuberculosis in Bamako were also MTB T1 (38.9%), *M. africanum* 2 (MAF2; 26.2%) and MTB LAM 10 (10.3%)^21^. Over the ten year surveillance period 50% of all cases of MDR-TB were associated with strains belonging to the T1^23^ reinforcing the previous findings that the T1 genotype was associated with MDR-TB in Bamako^21^.

Genomics has recently become an important tool in clinical management of TB cases^24–27^. By studying the genomes of TB isolates, we can infer resistance to anti-TB drugs, unravel transmission chains and gain insights into the evolutionary mechanisms that shape the epidemiology of TB^26,28–30^. Here, we present insights into the evolutionary mechanisms contributing to the rise of MDR-TB in Bamako. Although our sample size was limited, we tried to gain insights into the risk factors associated with MDR-TB in this setting using a logistic regression analysis. Our data highlights how the evolution of drug resistance hampers the fight against TB in a low-resource setting.

## Results

### Patient characteristics and genomic summary

A subset of randomly selected isolates from patients who had participated in study protocols at the University of Bamako were selected for whole genome sequencing (25). Isolates from 76 patients, who included 21 females and 55 males and individuals aged between 3 to 78 years, were analysed; these included 12 MDR-TB cases (Table 1). Most patients (n = 61, 80%) had not been previously treated for TB. While nine of the 15 retreatment cases (60%) had MDR-TB, only three among sixty-one new cases (5%) were MDR-TB. Sequencing reads mapped to the H37Rv reference genome with an average coverage of 33-fold (range 10 to 93-fold). *De novo* draft assemblies had an average of 4.4 million bases (range 4294898 to 5984674) and an average N50 of 57958 (range 26213 to 82606).

**Table 1:** A table summarizing the patient characteristics of the study participants. The table shows the distribution of HIV status, gender, treatment history and age.

		MDR	Non-MDR	Total (%)
Total		12	64	76 (100)
HIV Status	Negative	11	51	62 (82%)
	Positive	1	6	7 (9%)
	Not Done	0	7	7 (9%)
Gender	Female	2	19	21 (28%)
	Male	10	45	55 (72%)
Treatment History	New	3	58	61 (80%)
	Retreatment	9	6	15 (20%)
Age (years)	0–15	1	2	3 (4%)
	16–30	5	30	35 (46%)
	Above 30	6	32	38 (50%)

### Phylogeny

**Figure 1:**
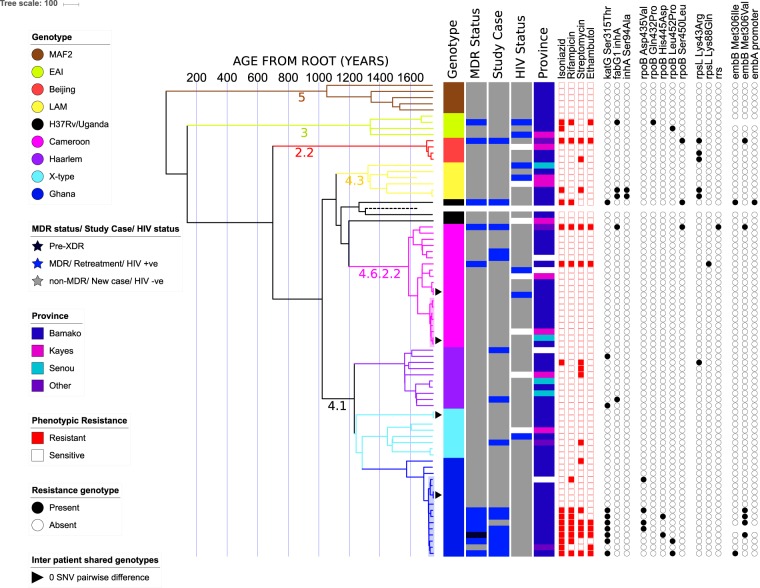
A time-dated phylogenetic tree of 76 MTBC isolates from pulmonary tuberculosis patients treated in Bamako, Mali between 2006 and 2013. Phylogenetic tree is shown alongside metadata including genotype, residential province, MDR-TB status based on phenotypic testing, HIV status and treatment history (new case or retreatment). The numbers on the x-axis show the number of years from the root. Closely related isolates from the Ghana genotype and Cameroon genotype are highlighted in blue and pink boxes respectively. White boxes in the metadata indicate missing data. The dashed line is the H37Rv reference genome. Branches are coloured based on genotype and labelled with SNP barcoding genotypes. Heat maps show phenotypic resistance and presence of resistance genotypes for first line drugs.

A time dated maximum likelihood phylogeny was reconstructed from 8508 variant core genome sites using BEAST. All the human-associated lineages of MTBC from the “modern” clade (Lineages 2,3 & 4) were detected among patients from Mali (Table 2). The dominant lineage was the Euro-American lineage, Lineage 4, and within Lineage 4 the Cameroon genotype was the most prevalent genotype (n = 20, 26%) (Fig. 1).

**Table 2:** A table showing the genotypic distribution of the isolates recovered from patients. The table shows the distribution of the major lineages of MTBC and the distribution of genotypes inferred from whole genome sequencing.

Lineage	Clade	MDR	Non-MDR	Total (%)
2	Beijing	1	3	4 (5.3)
3	EAI	1	3	4 (5.3)
4	Cameroon	2	18	20 (26.3)
	Ghana	7	9	16 (21.1)
	H37Rv	1	1	2 (2.6)
	Haarlem	0	10	10 (13.2)
	LAM	0	6	6 (7.9)
	Uganda	0	1	1 (1.3)
	X-type	0	8	8 (10.5)
6	MAF2	0	5	5 (6.6)
	Total	12	64	76

The Ghana genotype was the second most common genotype isolated from patients in the genomics subset. The Ghana genotype was represented by 16 isolates—seven of these were MDR-TB, therefore, the Ghana genotype represents over half of the 12 MDR-TB isolates in the genomics dataset. Patients infected with the Ghana genotype were over 4 times more likely to have MDR-TB than patients infected with other genotypes but the association was not significant after adjusting for retreatment cases and whether the patients resided in Bamako or not (OR 4.53, 95% CI 0.9–22.7, p value 0.067) (Table 3). Retreatment cases were 30 times more likely to have MDR-TB after adjusting for Ghana genotype and province of residence (OR 29.7, 95% CI 27.9–31.6, p value < 0.001).

**Table 3:** A summary of the MDR-TB risk factor analysis. The table shows the odds ratio, p-value and 95% confidence intervals for MDR-TB likelihood for patients infected with the Ghana genotype compared to other genotypes and retreatment cases compared to new cases.

Co-variates	Levels	MDR-TB count (%)	Odds ratio (95% CI)	p-value
Genotype	Others	5 (8.3)	—	
	Ghana	7 (43.8)	4.5 (0.9–22.7)	0.07
Treatment status	New case	3 (4.9)	—	
	Retreatment	9 (60)	108.7 (27.9–31.6)	<0.001

Other Euro-American genotypes included the LAM genotype (n = 6), two H37Rv-like isolates and one isolate from the Uganda genotype. Four isolates of the Beijing genotype from Lineage 2, including one MDR-TB, formed a monophyletic clade where isolates differed by an average of 23 core genome SNVs. Our estimates suggest that our Beijing genotype isolates shared a common recent ancestor 40 years ago. Five patients were infected with MAF2 strains, but none were MDR, and another four were infected with the East Asian Indian lineage strains including one MDR-TB retreatment case.

### Roles of the Ghana and Cameroon genotypes of MTBC

**Figure 2:**
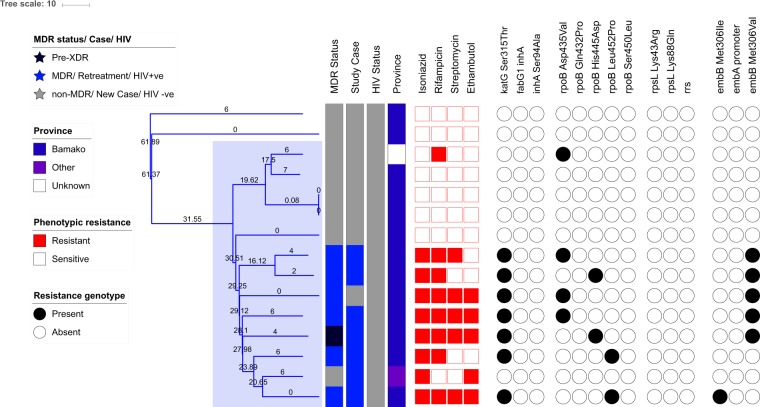
A time-dated phylogenetic tree of all Ghana genotype isolates in this dataset to highlight the evolution of MDR-TB in this lineage. Phylogeny shown alongside MDR status, treatment status, HIV status, phenotypic resistance profiles and resistance mutations for first line drugs are listed. Heat maps show phenotypic resistance and presence of resistance genotypes for first line drugs. Internal nodes are labelled with age in years.

Our phylogeny revealed a sub-clade of Ghana genotype isolates that diverged from a common recent ancestor approximately 30 years ago (95% HPD intervals 24.42–39.44) (Fig. 2). This sub-clade, which is highlighted in blue in Fig. 1, included seven MDR-TB and six non-MDR-TB isolates. On average, strains within this cluster differed by 12 core-genome SNVs. The most divergent pair of strains within this cluster differed by 24 core genome SNVs. The *katG* Ser315Thr isoniazid resistance mutation was conserved in all the isolates and in one non-MDR isoniazid resistant isolate. Three isoniazid resistant isolates also acquired the *fabG1 inhA* promoter-region mutation that is known to confer isoniazid and ethionamide resistance. MDR-TB isolates acquired rifampicin resistance through several different mutations in the *rpoB* gene (Fig. 2).

**Figure 3:**
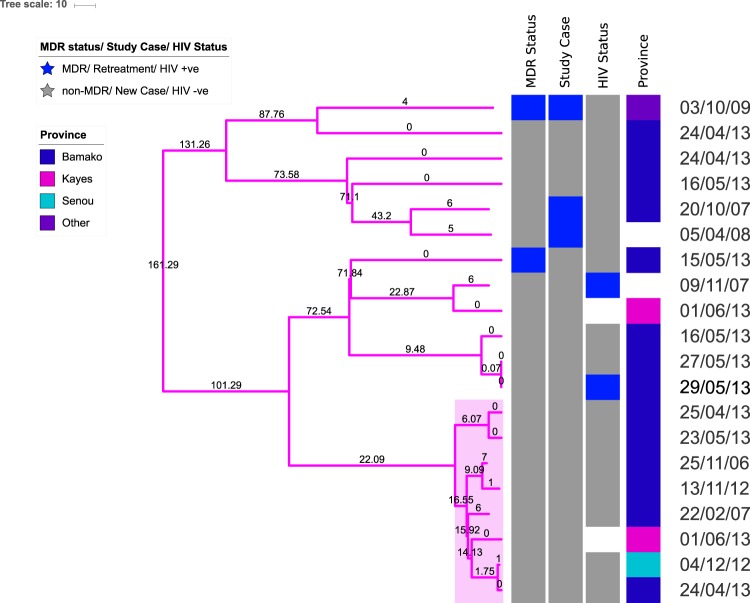
A time-dated phylogenetic tree of all Cameroon genotype isolates in our dataset. The phylogeny is shown alongside patient metadata and date of isolation to highlight the evolution of this emerging genotype in Bamako. Internal nodes are labelled with age in years.

Phylogenetic dating suggested that the Cameroon genotype isolates diverged from a common recent ancestor approximately 161 years ago (95% HPD intervals 129.52–195.39) to form three clusters (Fig. 3). The cluster of closely related genomes highlighted in pink in Fig. 1 was a sub-clade of the Cameroon genotype clade that encompassed eight non-MDR isolates. This sub clade was highly conserved and isolates within it differed by average pairwise distance of 8 core genome SNVs (range 0 to 13 SNVs). We estimated that this cluster emerged approximately 22 years ago (95% HPD intervals 15.32–29.61) with within-cluster internal node ages ranging from 1.75 years to 16.55 years (Fig. 3).

### Evidence of community transmission

**Figure 4:**
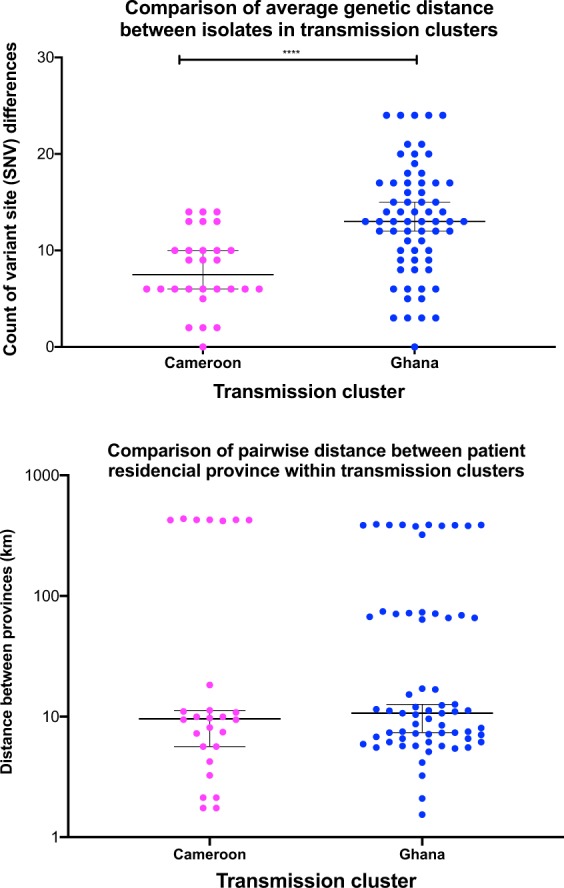
An illustration of the pairwise genetic distances between isolates and the pairwise distance between patients’ residential province within the potential transmission clusters. The lines on the charts show mean genetic distance and median distance respectively with error bars indicating the 95% confidence intervals.

Potential transmission clusters were identified as sub clades within the phylogeny that had an average pairwise genetic distance of 12 or less SNVs. Two clusters were identified, a Cameroon genotype cluster and a Ghana genotype cluster, which were illustrated by highlighting the subclades on the phylogenetic tree in Figs. 1–3. The average pairwise genetic differences in the Cameroon genotype (7 SNVs) cluster was significantly lower than the average distance in the Ghana genotype cluster (p value < 0.0001) (Fig. 4). The median pairwise distance between patients’ residential provinces as approximately 11 km in both clusters (Fig. 4) as most patients resided in provinces within Bamako.

Further evidence of community transmission was the identification of the same strain (0 SNV difference) from different patients on four occasions (marked in Fig. 1). In case 1 the strain was a Cameroon genotype strain, which was not from the highlighted transmission cluster. The strain was isolates from two patients in Bamako in 2013 residing in provinces 8 km apart. Case 2 also involved a Cameroon genotype strain, which was part of the transmission cluster. This strain was isolated from a patient in Senou 2012 and a second patient in Daoudabougou, Bamako in 2013 (~20 km apart). Moreover, this strain was closely related (2 SNV difference) to another strain isolated from a resident Kayes (~500 km from Bamako) in 2013. Case 3 involved a strain from the X-type genotype, which was found in two patients in residing in Bamako, Yirimadio and Kalaban-Coura (~ 10 km apart), in 2010. In case 4 the strain was within the Ghana genotype transmission cluster. Both patients, a male and a female aged 18 and 4 years respectively, were resident in Bamako in provinces 7.5 km apart and were both recruited in June 2013. This strain was closely related (3 SNV difference) to two other strains within the transmission cluster, one isolated from resident of Bamako in 2013 and the second one isolated in 2007 from a patient whose provincial domicile was not known.

## Discussion

Estimating the burden of drug resistant tuberculosis in Mali has been challenging due to political instability and weak infrastructure. Laboratory based drug resistance surveillance carried out in Bamako between 2006 and 2014 showed that the levels of MDR-TB among patients has remained constant and relatively high^22^. Our study employs whole genome sequencing to study the evolutionary mechanisms driving the rise of MDR-TB in Mali in recent times and the emergence of virulent lineages of MTBC using a subset of isolates reported in Gehre *et al*. (25).

Our data shows that an important driver of MDR-TB in Bamako is a sub-clade of the Ghana genotype, which often has spoligo typing patters belonging to the T1 family. This family has previously been implicated with a higher likelihood of resistance compared to other genotypes^21^. The MDR-TB Ghana genotype sub-clade emerged approximately 30 years ago, most likely through the initial acquisition on the *katG* Ser415Thr isoniazid resistant mutation. Isoniazid resistance probably presented a selective advantage to the ancestral clone, allowing it to form divergent sub-clones that acquired rifampicin resistance through different mechanisms and is now the leading cause of MDR-TB in Bamako.

The main genotypes of MTBC known to cause tuberculosis in Bamako are the T1 family, Cameroon genotype (LAM10) and the *M. Africanum* West Africa 2 lineage strains^21,23^. Our data suggests that the Cameroon genotype is on the rise in Bamako compared to recent estimates^21^ and there is evidence of community transmission. Although the emerging Cameroon genotype strains are non MDR-TB, the situation should be carefully monitored. In Nigeria, a neighbouring West African country, we have seen evidence of widespread MDR-TB within the Cameroon genotype and signs of community transmission^31^.

The first report of Beijing strains causing tuberculosis in Bamako was in 2008 when unrelated strains were detected in two patients with active pulmonary tuberculosis^32^. Here we identified a clade of related Beijing isolates that differed on average by 22 SNVs (range 12 to 30) including one MDR-TB isolate. The Beijing genotype has emerged around the world^33,34^ and has demonstrated the ability to spread rapidly once introduced into a new population^35^. Bioinformatics approaches have identified mutations in virulence genes that are unique to the Beijing genotype^36^ and mutations in immune response associated genes that may aid host-pathogen co-evolution to the detriment of TB control^37^. The Beijing genotype has also been linked to MDR-TB in other settings^38^. Tuberculosis surveillance in Mali would be helpful in identifying the extent to which the Beijing and other virulent genotypes have spread in the community.

A disproportionate number of MDR-TB cases end up in retreatment or treatment failure. This is because in Bamako only retreatment or treatment failure patients were subjected to drug susceptibility testing at the National Tuberculosis Reference Laboratory. New cases with MDR-TB would not be detected unless they were enrolled in research protocols, at the SEREFO program where all isolates undergo routine phenotypic or genotypic susceptibility testing^22^. Implementing drug susceptibility testing across all public hospitals is not cost-effective but the Hain *MTBDRplus* Line Probe Assay could provide an effective screening tool to detect the most common forms of isoniazid and rifampicin resistance.

## Conclusion

MDR-TB is a growing public health concern in Bamako. MDR-TB in Bamako is often caused by a 30-year old sub clade of the Ghana genotype. Introducing molecular susceptibility testing as a screening tool in public hospitals will improve the early detection of MDR-TB, improve treatment outcomes and curb the spread of the MDR-TB including the Ghana genotype MDR-TB sub clade.

## Methods

### Demographics of study area

The study participants from Mali were recruited in the capital city Bamako, where according to the Ministry of Health more than a third of all TB cases in Mali were managed in 2014^39^. Bamako is divided into six districts and each district has a TB referral health centre that is equipped with diagnosis and treatment facilities. Our isolates were collected at the University Teaching Hospital as part of research protocols, which acts as the principal TB referral centre. Retreatment cases from across Mali are referred to the University Teaching Hospital for further investigation and treatment.

### Ethical approval and informed consent

All study protocols were approved by the ethic committee of the faculty of medicine, pharmacy and dentistry of Bamako as well as the MRC Unit The Gambia at LSHTM and Gambia Government joint ethics committee. Signed informed consent was obtained from all patients and from the legal guardian for patients under 18 years of age. All methods were carried out in accordance with relevant guidelines and regulations.

### Study isolates

In a surveillance of tuberculosis between 2006 and 2014, which included 522 patients treated in Bamako, co-author Diarra and colleagues^22^. From this dataset a subset of 208 isolates were sent to MRC Unit The Gambia at LSHTM, as part of the WANETAM survey on drug resistant tuberculosis in West Africa^20^. This is an ad hoc dataset whereby 85 isolates were chosen at random without any set criteria.

Sub cultured mycobacterial isolates were sent to the MRC Unit The Gambia at LSHTM for viability and antimicrobial susceptibility testing. Isolates were sub-cultured onto MGIT 960 system (Becton Dickinson, Oxford Science Park, Oxford, UK) for viability testing. Positive cultures were tested for purity by inoculation on blood agar and followed up only if there was no growth after 24 hours incubation. The presence of MTBC was confirmed by Ziehl–Neelsen staining. Susceptibility testing for the first-line drugs was performed on the MGIT 960 system (Becton Dickinson, Oxford Science Park, Oxford, UK) according to manufacturer’s instructions^40^: streptomycin (STR, 1 µg/mL), isoniazid (INH, 0.1 µg/mL), rifampicin (RIF, 1 µg/mL), and ethambutol (EMB, 4.5 µg/mL). Multidrug-resistant strains (resistant to isoniazid and rifampicin) were further tested for susceptibility to the second-line drugs capreomycin (CAP, 2.5 µg/mL), ofloxacin (OFX, 2 µg/mL), and ethionamide (ETH, 5 µg/mL) (Sigma-Aldrich, St. Louis, Mo, USA)^20^. The MRC Unit The Gambia at LSHTM TB Diagnostics Laboratory participates in external quality assurance from the National External Quality Assessment Service (NEQAS), UK (http://www.ukneqas.org.uk/) and is ISO15189:2012 accredited for’ first- and second-line DST together with other TB diagnostic assays.

### Whole genome sequencing

In order to extract whole-genomic DNA isolates were grown in Middlebrooks 7H9 liquid medium (Sigma Aldrich, Gillingham, Dorset, UK) for 3–8 weeks and the presence of MTBC was confirmed by the rapid test Capilia^TM^ TB-Neo (Sigma Aldrich, Gillingham, Dorset, UK) or BD MGIT™ TBc (Becton, Dickinson and Company, Oxford Science Park, Oxford, UK). To test for purity the culture medium was streaked onto blood agar and incubated at 37 °C for 48 hours. Finally genomic DNA extraction was performed using the cetyl trimethylammonium bromide (CTAB) method^41^. Genomic DNA libraries were prepared for whole genome sequencing using the Nextera XT library prep kit according to manufacturer’s instructions (Illumina, Little Chesterford, UK) and sequenced on an Illumina MiSeq following the manufacturer’s instructions (Illumina, Little Chesterford, UK).

### Genomic and phylogenetic analysis

An adhoc subset of eighty-five isolates of MTBC were selected from 208 archived Malian isolates for whole-genome sequencing. Seven isolates that had low coverage (<10X) and two isolates that had no patient metadata or antimicrobial susceptibility profiles were excluded from the analysis. Sequencing reads were uploaded on the PhyResSe webtool for lineage assignment and antimicrobial susceptibility prediction^29^. Phylogenetic lineages were inferred based on the whole genome single nucleotide polymorphism typing scheme described by Homolka and colleagues^42^.

For each isolate *de novo* contigs were generated from the paired-end sequencing reads using SPAdes (kmers: 21, 33, 55, 77, 99 and 127)^43^. To correct assemblies, SOAPaligner v2.21 was used to to remap sequencing reads onto each assembly and bases in the assembly were filtered based on a quality score <20 and <10 reads mapped. Each assembly was mapped to the H37Rv reference genome using the nucmer function in MUMmer^44^ to generate a consensus sequence and all concensus sequences were compiled in a multiple sequence alignment with the reference genome. Using custom Perl scripts all repeat regions identified by Sergeant *et al*.^45^ were removed from the alignment and the core genome was inferred from the multiple sequence alignment as sites conserved in all isolates^46,47^.

Single nucleotide variants (SNVs) were extracted from the core genome multiple sequence alignment using custom Perl scripts and were uploaded on the Bayesian Evolutionary Analysis Sampling Trees (BEAST Geo Shere package version 1.1.2) tool^48^. A time dated maximum likelihood phylogeny was reconstructed from variable sites in the core genome using a relaxed clock gamma time reversible model with substitution rates estimated from the sequence alignment data and a Yule model for birth rate. Date of sample collection (year and month of collection) and GPS coordinates for patients’ residential province were uploaded as traits. The phylogenetic tree was visualised in the Interactive Tree of Life (iTol)^49^ and FigTree (Version 1.4.2) and annotated using Inkscape^50^. A multifasta containing variant core genome sites was uploaded on MEGA 7 and a distant matrix was generated based on the pairwise number of differences between isolates.

### MDR-TB risk factor analysis

The associated metadata for each patient was uploaded onto Stata version 12.0 for statistical analysis. A logistic regression analysis using the Chi square test was performed to test whether isolates belonging to the Ghana genotype were more likely to be MDR-TB than isolates belonging to other lineages. We controlled for whether the patients were retreatment cases of new cases and error bars were adjusted for based on the province of origin of the patient.

### Study limitations

The limitation of the study is that patients were recruited from the University teaching hospital, which is a referral center. The prevalence of genotypes in our dataset may not be an exact representation of the prevalence of genotypes in the communities within Bamako.

### Data declaration

The sequencing reads generated for the analysis presented in this paper have been uploaded onto the European Nucleotide Archives under the project accession number PRJEB27446.
